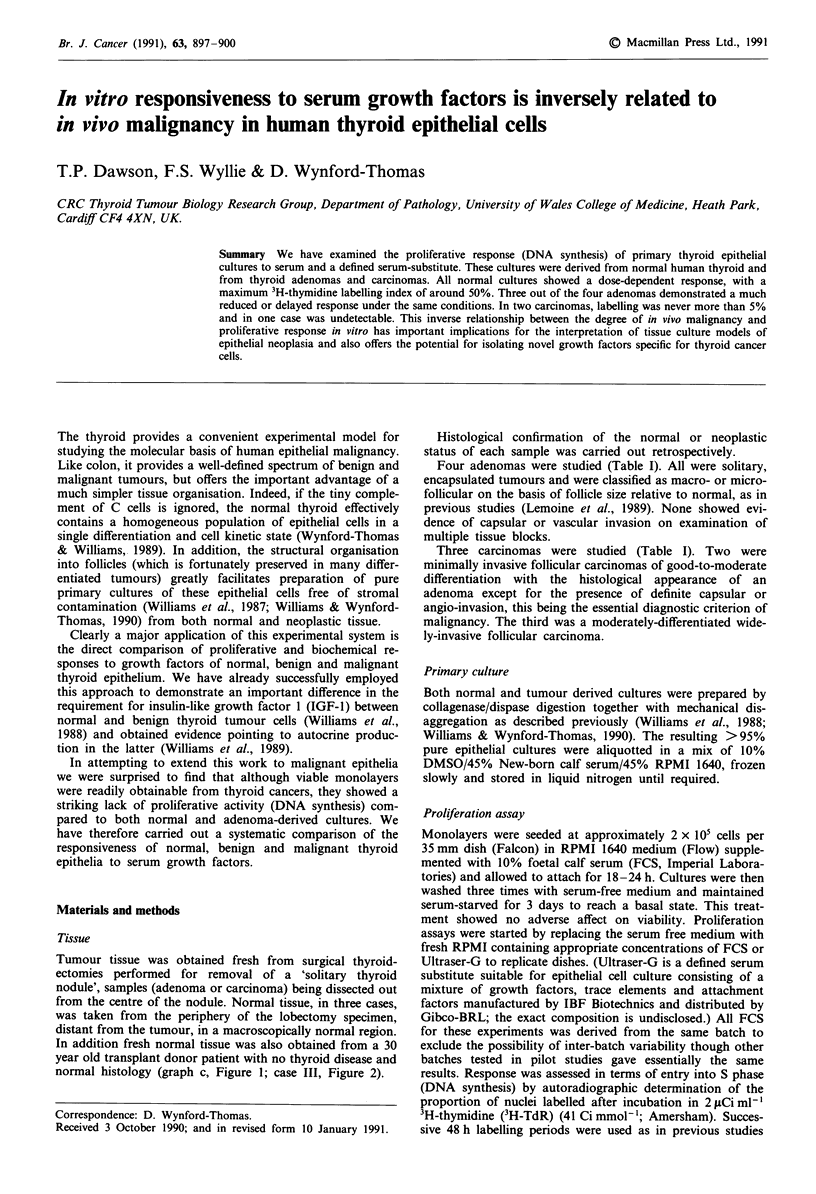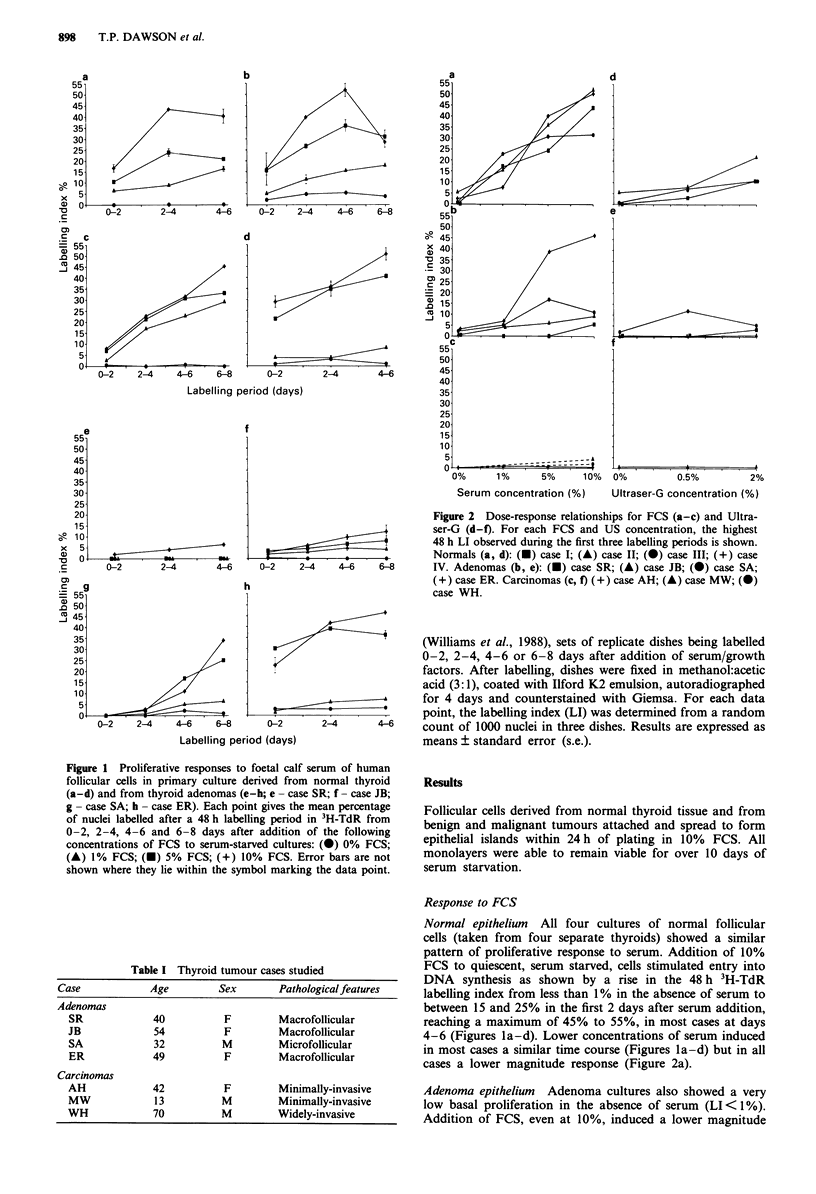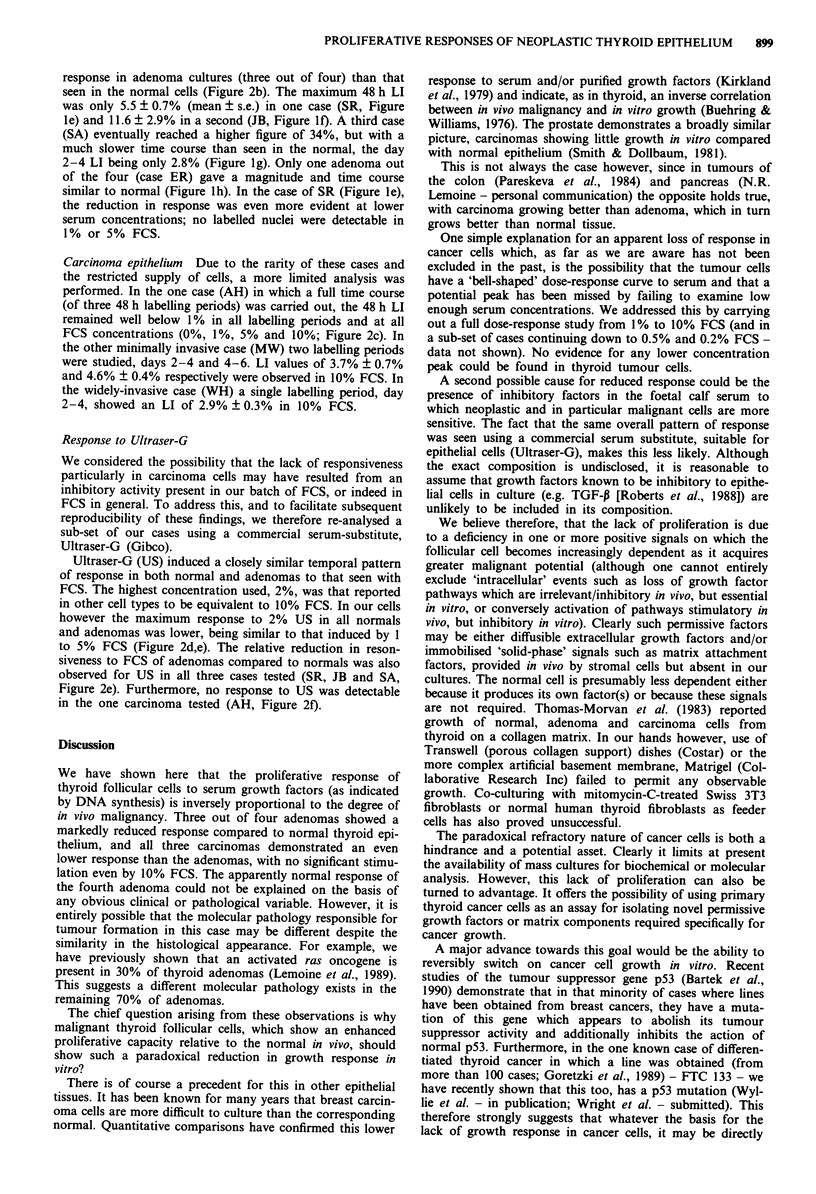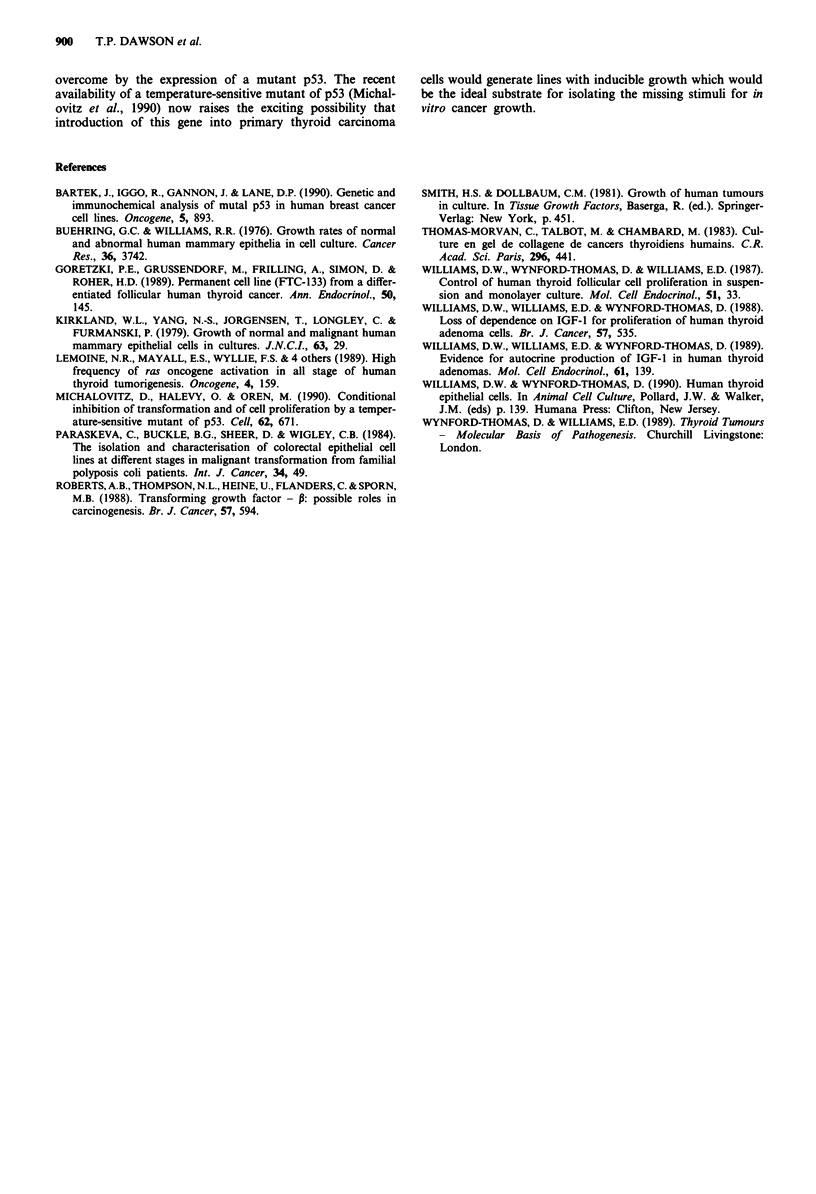# In vitro responsiveness to serum growth factors is inversely related to in vivo malignancy in human thyroid epithelial cells.

**DOI:** 10.1038/bjc.1991.197

**Published:** 1991-06

**Authors:** T. P. Dawson, F. S. Wyllie, D. Wynford-Thomas

**Affiliations:** Department of Pathology, University of Wales College of Medicine, Heath Park, Cardiff, UK.

## Abstract

We have examined the proliferative response (DNA synthesis) of primary thyroid epithelial cultures to serum and a defined serum-substitute. These cultures were derived from normal human thyroid and from thyroid adenomas and carcinomas. All normal cultures showed a dose-dependent response, with a maximum 3H-thymidine labelling index of around 50%. Three out of the four adenomas demonstrated a much reduced or delayed response under the same conditions. In two carcinomas, labelling was never more than 5% and in one case was undetectable. This inverse relationship between the degree of in vivo malignancy and proliferative response in vitro has important implications for the interpretation of tissue culture models of epithelial neoplasia and also offers the potential for isolating novel growth factors specific for thyroid cancer cells.


Br. J. Cancer (1991), 63, 897-900

© Macmillan Press Ltd., 1991

### *In vitro* responsiveness to serum growth factors is inversely related to *in vivo* malignancy in human thyroid epithelial cells

T.P. Dawson, F.S. Wyllie & D. Wynford-Thomas

*CRC Thyroid Tumour Biology Research Group, Department of Pathology, University of Wales College of Medicine, Heath Park, Cardiff CF4 4XN, UK.*

**Summary** We have examined the proliferative response (DNA synthesis) of primary thyroid epithelial cultures to serum and a defined serum-substitute. These cultures were derived from normal human thyroid and from thyroid adenomas and carcinomas. All normal cultures showed a dose-dependent response, with a maximum $^{3}$H-thymidine labelling index of around 50%. Three out of the four adenomas demonstrated a much reduced or delayed response under the same conditions. In two carcinomas, labelling was never more than 5% and in one case was undetectable. This inverse relationship between the degree of *in vivo* malignancy and proliferative response *in vitro* has important implications for the interpretation of tissue culture models of epithelial neoplasia and also offers the potential for isolating novel growth factors specific for thyroid cancer cells.

The thyroid provides a convenient experimental model for studying the molecular basis of human epithelial malignancy. Like colon, it provides a well-defined spectrum of benign and malignant tumours, but offers the important advantage of a much simpler tissue organisation. Indeed, if the tiny complement of C cells is ignored, the normal thyroid effectively contains a homogeneous population of epithelial cells in a single differentiation and cell kinetic state (Wynford-Thomas & Williams, 1989). In addition, the structural organisation into follicles (which is fortunately preserved in many differentiated tumours) greatly facilitates preparation of pure primary cultures of these epithelial cells free of stromal contamination (Williams *et al.*, 1987; Williams & Wynford-Thomas, 1990) from both normal and neoplastic tissue.

Clearly a major application of this experimental system is the direct comparison of proliferative and biochemical responses to growth factors of normal, benign and malignant thyroid epithelium. We have already successfully employed this approach to demonstrate an important difference in the requirement for insulin-like growth factor 1 (IGF-1) between normal and benign thyroid tumour cells (Williams *et al.*, 1988) and obtained evidence pointing to autocrine production in the latter (Williams *et al.*, 1989).

In attempting to extend this work to malignant epithelia we were surprised to find that although viable monolayers were readily obtainable from thyroid cancers, they showed a striking lack of proliferative activity (DNA synthesis) compared to both normal and adenoma-derived cultures. We have therefore carried out a systematic comparison of the responsiveness of normal, benign and malignant thyroid epithelia to serum growth factors.

### Materials and methods

### *Tissue*

Tumour tissue was obtained fresh from surgical thyroidectomies performed for removal of a 'solitary thyroid nodule', samples (adenoma or carcinoma) being dissected out from the centre of the nodule. Normal tissue, in three cases, was taken from the periphery of the lobectomy specimen, distant from the tumour, in a macroscopically normal region. In addition fresh normal tissue was also obtained from a 30 year old transplant donor patient with no thyroid disease and normal histology (graph c, Figure 1; case III, Figure 2).

Correspondence: D. Wynford-Thomas.
Received 3 October 1990; and in revised form 10 January 1991.

Histological confirmation of the normal or neoplastic status of each sample was carried out retrospectively.

Four adenomas were studied (Table I). All were solitary, encapsulated tumours and were classified as macro- or micro-follicular on the basis of follicle size relative to normal, as in previous studies (Lemoine *et al.*, 1989). None showed evidence of capsular or vascular invasion on examination of multiple tissue blocks.

Three carcinomas were studied (Table I). Two were minimally invasive follicular carcinomas of good-to-moderate differentiation with the histological appearance of an adenoma except for the presence of definite capsular or angio-invasion, this being the essential diagnostic criterion of malignancy. The third was a moderately-differentiated widely-invasive follicular carcinoma.

### *Primary culture*

Both normal and tumour derived cultures were prepared by collagenase/dispase digestion together with mechanical disaggregation as described previously (Williams *et al.*, 1988; Williams & Wynford-Thomas, 1990). The resulting >95% pure epithelial cultures were aliquotted in a mix of 10% DMSO/45% New-born calf serum/45% RPMI 1640, frozen slowly and stored in liquid nitrogen until required.

### *Proliferation assay*

Monolayers were seeded at approximately $2 \times 10^5$ cells per 35 mm dish (Falcon) in RPMI 1640 medium (Flow) supplemented with 10% foetal calf serum (FCS, Imperial Laboratories) and allowed to attach for 18-24 h. Cultures were then washed three times with serum-free medium and maintained serum-starved for 3 days to reach a basal state. This treatment showed no adverse affect on viability. Proliferation assays were started by replacing the serum free medium with fresh RPMI containing appropriate concentrations of FCS or Ultraser-G to replicate dishes. (Ultraser-G is a defined serum substitute suitable for epithelial cell culture consisting of a mixture of growth factors, trace elements and attachment factors manufactured by IBF Biotechnics and distributed by Gibco-BRL; the exact composition is undisclosed.) All FCS for these experiments was derived from the same batch to exclude the possibility of inter-batch variability though other batches tested in pilot studies gave essentially the same results. Response was assessed in terms of entry into S phase (DNA synthesis) by autoradiographic determination of the proportion of nuclei labelled after incubation in 2 $\mu$Ci ml$^{-1}$ $^{3}$H-thymidine ($^{3}$H-TdR) (41 Ci mmol$^{-1}$; Amersham). Successive 48 h labelling periods were used as in previous studies

**Figure 1** Proliferative responses to foetal calf serum of human follicular cells in primary culture derived from normal thyroid (**a–d**) and from thyroid adenomas (**e–h**; **e** – case SR; **f** – case JB; **g** – case SA; **h** – case ER). Each point gives the mean percentage of nuclei labelled after a 48 h labelling period in $^3$H-TdR from 0–2, 2–4, 4–6 and 6–8 days after addition of the following concentrations of FCS to serum-starved cultures: (●) 0% FCS; (▲) 1% FCS; (■) 5% FCS; (+) 10% FCS. Error bars are not shown where they lie within the symbol marking the data point.

**Table I** Thyroid tumour cases studied

| *Case* | *Age* | *Sex* | *Pathological features* |
|---|---|---|---|
| *Adenomas* | | | |
| SR | 40 | F | Macrofollicular |
| JB | 54 | F | Macrofollicular |
| SA | 32 | M | Microfollicular |
| ER | 49 | F | Macrofollicular |
| *Carcinomas* | | | |
| AH | 42 | F | Minimally-invasive |
| MW | 13 | M | Minimally-invasive |
| WH | 70 | M | Widely-invasive |

**Figure 2** Dose-response relationships for FCS (**a–c**) and Ultraser-G (**d–f**). For each FCS and US concentration, the highest 48 h LI observed during the first three labelling periods is shown. Normals (**a, d**): (■) case I; (▲) case II; (●) case III; (+) case IV. Adenomas (**b, e**): (■) case SR; (▲) case JB; (●) case SA; (+) case ER. Carcinomas (**c, f**) (+) case AH; (▲) case MW; (●) case WH.

(Williams *et al.*, 1988), sets of replicate dishes being labelled 0–2, 2–4, 4–6 or 6–8 days after addition of serum/growth factors. After labelling, dishes were fixed in methanol:acetic acid (3:1), coated with Ilford K2 emulsion, autoradiographed for 4 days and counterstained with Giemsa. For each data point, the labelling index (LI) was determined from a random count of 1000 nuclei in three dishes. Results are expressed as means ± standard error (s.e.).

### Results

Follicular cells derived from normal thyroid tissue and from benign and malignant tumours attached and spread to form epithelial islands within 24 h of plating in 10% FCS. All monolayers were able to remain viable for over 10 days of serum starvation.

### *Response to FCS*

*Normal epithelium* All four cultures of normal follicular cells (taken from four separate thyroids) showed a similar pattern of proliferative response to serum. Addition of 10% FCS to quiescent, serum starved, cells stimulated entry into DNA synthesis as shown by a rise in the 48 h $^3$H-TdR labelling index from less than 1% in the absence of serum to between 15 and 25% in the first 2 days after serum addition, reaching a maximum of 45% to 55%, in most cases at days 4–6 (Figures 1a–d). Lower concentrations of serum induced in most cases a similar time course (Figures 1a–d) but in all cases a lower magnitude response (Figure 2a).

*Adenoma epithelium* Adenoma cultures also showed a very low basal proliferation in the absence of serum (LI < 1%). Addition of FCS, even at 10%, induced a lower magnitude response in adenoma cultures (three out of four) than that seen in the normal cells (Figure 2b). The maximum 48 h LI was only 5.5 ± 0.7% (mean ± s.e.) in one case (SR, Figure 1e) and 11.6 ± 2.9% in a second (JB, Figure 1f). A third case (SA) eventually reached a higher figure of 34%, but with a much slower time course than seen in the normal, the day 2–4 LI being only 2.8% (Figure 1g). Only one adenoma out of the four (case ER) gave a magnitude and time course similar to normal (Figure 1h). In the case of SR (Figure 1e), the reduction in response was even more evident at lower serum concentrations; no labelled nuclei were detectable in 1% or 5% FCS.

*Carcinoma epithelium* Due to the rarity of these cases and the restricted supply of cells, a more limited analysis was performed. In the one case (AH) in which a full time course (of three 48 h labelling periods) was carried out, the 48 h LI remained well below 1% in all labelling periods and at all FCS concentrations (0%, 1%, 5% and 10%; Figure 2c). In the other minimally invasive case (MW) two labelling periods were studied, days 2–4 and 4–6. LI values of 3.7% ± 0.7% and 4.6% ± 0.4% respectively were observed in 10% FCS. In the widely-invasive case (WH) a single labelling period, day 2–4, showed an LI of 2.9% ± 0.3% in 10% FCS.

*Response to Ultraser-G*

We considered the possibility that the lack of responsiveness particularly in carcinoma cells may have resulted from an inhibitory activity present in our batch of FCS, or indeed in FCS in general. To address this, and to facilitate subsequent reproducibility of these findings, we therefore re-analysed a sub-set of our cases using a commercial serum-substitute, Ultraser-G (Gibco).

Ultraser-G (US) induced a closely similar temporal pattern of response in both normal and adenomas to that seen with FCS. The highest concentration used, 2%, was that reported in other cell types to be equivalent to 10% FCS. In our cells however the maximum response to 2% US in all normals and adenomas was lower, being similar to that induced by 1 to 5% FCS (Figure 2d,e). The relative reduction in resonsiveness to FCS of adenomas compared to normals was also observed for US in all three cases tested (SR, JB and SA, Figure 2e). Furthermore, no response to US was detectable in the one carcinoma tested (AH, Figure 2f).

### Discussion

We have shown here that the proliferative response of thyroid follicular cells to serum growth factors (as indicated by DNA synthesis) is inversely proportional to the degree of *in vivo* malignancy. Three out of four adenomas showed a markedly reduced response compared to normal thyroid epithelium, and all three carcinomas demonstrated an even lower response than the adenomas, with no significant stimulation even by 10% FCS. The apparently normal response of the fourth adenoma could not be explained on the basis of any obvious clinical or pathological variable. However, it is entirely possible that the molecular pathology responsible for tumour formation in this case may be different despite the similarity in the histological appearance. For example, we have previously shown that an activated *ras* oncogene is present in 30% of thyroid adenomas (Lemoine *et al.*, 1989). This suggests a different molecular pathology exists in the remaining 70% of adenomas.

The chief question arising from these observations is why malignant thyroid follicular cells, which show an enhanced proliferative capacity relative to the normal *in vivo*, should show such a paradoxical reduction in growth response *in vitro*?

There is of course a precedent for this in other epithelial tissues. It has been known for many years that breast carcinoma cells are more difficult to culture than the corresponding normal. Quantitative comparisons have confirmed this lower response to serum and/or purified growth factors (Kirkland *et al.*, 1979) and indicate, as in thyroid, an inverse correlation between *in vivo* malignancy and *in vitro* growth (Buehring & Williams, 1976). The prostate demonstrates a broadly similar picture, carcinomas showing little growth *in vitro* compared with normal epithelium (Smith & Dollbaum, 1981).

This is not always the case however, since in tumours of the colon (Pareskeva *et al.*, 1984) and pancreas (N.R. Lemoine – personal communication) the opposite holds true, with carcinoma growing better than adenoma, which in turn grows better than normal tissue.

One simple explanation for an apparent loss of response in cancer cells which, as far as we are aware has not been excluded in the past, is the possibility that the tumour cells have a 'bell-shaped' dose-response curve to serum and that a potential peak has been missed by failing to examine low enough serum concentrations. We addressed this by carrying out a full dose-response study from 1% to 10% FCS (and in a sub-set of cases continuing down to 0.5% and 0.2% FCS – data not shown). No evidence for any lower concentration peak could be found in thyroid tumour cells.

A second possible cause for reduced response could be the presence of inhibitory factors in the foetal calf serum to which neoplastic and in particular malignant cells are more sensitive. The fact that the same overall pattern of response was seen using a commercial serum substitute, suitable for epithelial cells (Ultraser-G), makes this less likely. Although the exact composition is undisclosed, it is reasonable to assume that growth factors known to be inhibitory to epithelial cells in culture (e.g. TGF-$\beta$ [Roberts *et al.*, 1988]) are unlikely to be included in its composition.

We believe therefore, that the lack of proliferation is due to a deficiency in one or more positive signals on which the follicular cell becomes increasingly dependent as it acquires greater malignant potential (although one cannot entirely exclude 'intracellular' events such as loss of growth factor pathways which are irrelevant/inhibitory *in vivo*, but essential *in vitro*, or conversely activation of pathways stimulatory *in vivo*, but inhibitory *in vitro*). Clearly such permissive factors may be either diffusible extracellular growth factors and/or immobilised 'solid-phase' signals such as matrix attachment factors, provided *in vivo* by stromal cells but absent in our cultures. The normal cell is presumably less dependent either because it produces its own factor(s) or because these signals are not required. Thomas-Morvan *et al.* (1983) reported growth of normal, adenoma and carcinoma cells from thyroid on a collagen matrix. In our hands however, use of Transwell (porous collagen support) dishes (Costar) or the more complex artificial basement membrane, Matrigel (Collaborative Research Inc) failed to permit any observable growth. Co-culturing with mitomycin-C-treated Swiss 3T3 fibroblasts or normal human thyroid fibroblasts as feeder cells has also proved unsuccessful.

The paradoxical refractory nature of cancer cells is both a hindrance and a potential asset. Clearly it limits at present the availability of mass cultures for biochemical or molecular analysis. However, this lack of proliferation can also be turned to advantage. It offers the possibility of using primary thyroid cancer cells as an assay for isolating novel permissive growth factors or matrix components required specifically for cancer growth.

A major advance towards this goal would be the ability to reversibly switch on cancer cell growth *in vitro*. Recent studies of the tumour suppressor gene p53 (Bartek *et al.*, 1990) demonstrate that in that minority of cases where lines have been obtained from breast cancers, they have a mutation of this gene which appears to abolish its tumour suppressor activity and additionally inhibits the action of normal p53. Furthermore, in the one known case of differentiated thyroid cancer in which a line was obtained (from more than 100 cases; Goretzki *et al.*, 1989) – FTC 133 – we have recently shown that this too, has a p53 mutation (Wyllie *et al.* – in publication; Wright *et al.* – submitted). This therefore strongly suggests that whatever the basis for the lack of growth response in cancer cells, it may be directly overcome by the expression of a mutant p53. The recent availability of a temperature-sensitive mutant of p53 (Michalovitz *et al.*, 1990) now raises the exciting possibility that introduction of this gene into primary thyroid carcinoma cells would generate lines with inducible growth which would be the ideal substrate for isolating the missing stimuli for *in vitro* cancer growth.

### References

BARTEK, J., IGGO, R., GANNON, J. & LANE, D.P. (1990). Genetic and immunochemical analysis of mutal p53 in human breast cancer cell lines. *Oncogene*, **5**, 893.

BUEHRING, G.C. & WILLIAMS, R.R. (1976). Growth rates of normal and abnormal human mammary epithelia in cell culture. *Cancer Res.*, **36**, 3742.

GORETZKI, P.E., GRUSSENDORF, M., FRILLING, A., SIMON, D. & ROHER, H.D. (1989). Permanent cell line (FTC-133) from a differentiated follicular human thyroid cancer. *Ann. Endocrinol.*, **50**, 145.

KIRKLAND, W.L., YANG, N.-S., JORGENSEN, T., LONGLEY, C. & FURMANSKI, P. (1979). Growth of normal and malignant human mammary epithelial cells in cultures. *J.N.C.I.*, **63**, 29.

LEMOINE, N.R., MAYALL, E.S., WYLLIE, F.S. & 4 others (1989). High frequency of *ras* oncogene activation in all stage of human thyroid tumorigenesis. *Oncogene*, **4**, 159.

MICHALOVITZ, D., HALEVY, O. & OREN, M. (1990). Conditional inhibition of transformation and of cell proliferation by a temperature-sensitive mutant of p53. *Cell*, **62**, 671.

PARASKEVA, C., BUCKLE, B.G., SHEER, D. & WIGLEY, C.B. (1984). The isolation and characterisation of colorectal epithelial cell lines at different stages in malignant transformation from familial polyposis coli patients. *Int. J. Cancer*, **34**, 49.

ROBERTS, A.B., THOMPSON, N.L., HEINE, U., FLANDERS, C. & SPORN, M.B. (1988). Transforming growth factor – $\beta$: possible roles in carcinogenesis. *Br. J. Cancer*, **57**, 594.

SMITH, H.S. & DOLLBAUM, C.M. (1981). Growth of human tumours in culture. In *Tissue Growth Factors*, Baserga, R. (ed.). Springer-Verlag: New York, p. 451.

THOMAS-MORVAN, C., TALBOT, M. & CHAMBARD, M. (1983). Culture en gel de collagene de cancers thyroidiens humains. *C.R. Acad. Sci. Paris*, **296**, 441.

WILLIAMS, D.W., WYNFORD-THOMAS, D. & WILLIAMS, E.D. (1987). Control of human thyroid follicular cell proliferation in suspension and monolayer culture. *Mol. Cell Endocrinol.*, **51**, 33.

WILLIAMS, D.W., WILLIAMS, E.D. & WYNFORD-THOMAS, D. (1988). Loss of dependence on IGF-1 for proliferation of human thyroid adenoma cells. *Br. J. Cancer*, **57**, 535.

WILLIAMS, D.W., WILLIAMS, E.D. & WYNFORD-THOMAS, D. (1989). Evidence for autocrine production of IGF-1 in human thyroid adenomas. *Mol. Cell Endocrinol.*, **61**, 139.

WILLIAMS, D.W. & WYNFORD-THOMAS, D. (1990). Human thyroid epithelial cells. In *Animal Cell Culture*, Pollard, J.W. & Walker, J.M. (eds) p. 139. Humana Press: Clifton, New Jersey.

WYNFORD-THOMAS, D. & WILLIAMS, E.D. (1989). *Thyroid Tumours – Molecular Basis of Pathogenesis*. Churchill Livingstone: London.